# Coupled effects of oil spill and hurricane on saltmarsh terrestrial arthropods

**DOI:** 10.1371/journal.pone.0194941

**Published:** 2018-04-11

**Authors:** Wokil Bam, Linda M. Hooper-Bui, Rachel M. Strecker, Puspa L. Adhikari, Edward B. Overton

**Affiliations:** 1 Department of Oceanography and Coastal Sciences, Louisiana State University, Baton Rouge, United States of America; 2 Department of Environmental Sciences, Louisiana State University, Baton Rouge, United States of America; Centro de Investigacion Cientifica y de Educacion Superior de Ensenada Division de Fisica Aplicada, MEXICO

## Abstract

Terrestrial arthropods play an important role in saltmarsh ecosystems, mainly affecting the saltmarsh’s primary production as the main consumers of terrestrial primary production and decomposition. Some of these arthropods, including selected insects and spiders, can be used as ecological indicators of overall marsh environmental health, as they are differentially sensitive to ecological stressors, such as land loss, erosion, oil spills, and tropical storms. In the present study, we used terrestrial arthropods collected from seven (three lightly-oiled, four heavily-oiled) sites in Barataria Bay and from three unoiled reference sites in Delacroix, Louisiana, to determine the impacts of the distribution and re-distribution of *Deepwater Horizon* (DWH) oil on these saltmarsh ecosystems. A total of 9,476 and 12,256 insects were collected in 2013 and 2014, respectively. The results show that the terrestrial arthropods were negatively affected by the re-distribution of DWH oil by Hurricane Isaac in 2012, although the level of impacts varied among the arthropod groups. Moreover, the mean diversity index was higher (>1.5) in 2014 than in 2013 (<1.5) for all sites, suggesting a recovery trajectory of the saltmarsh arthropod population. The higher taxonomic richness observed in the reference sites compared to the oiled sites for both years also indicated long-term impacts of DWH oil to the saltmarsh arthropod community. Whereas a slow recovery of certain terrestrial arthropods was observed, long-term monitoring of arthropod communities would help better understand the recovery and succession of the marsh ecosystems.

## Introduction

Saltmarshes play an integral part in many coastal ecosystems, which support diverse communities of fish, birds, benthic invertebrates, and a large number of terrestrial arthropods. Terrestrial arthropods are critical to the saltmarsh ecosystem, serving as primary, secondary, and tertiary consumers, as well as decomposers [1]. They are one of the major food sources for saltmarsh birds and estuarine fishes, thus providing an important link among the different trophic levels in the saltmarsh [2–4]. Certain taxa, such as ants, spiders, and beetles, are very sensitive to changes in the environment and can be used as ecological indicators of the overall health of saltmarshes [5–7]. Due to short-term life cycle and reproductive times, most arthropods are considered to be ideal indicators for monitoring the ecosystem for both short-term and long-term control [8,9]. The large population size, high reproductive rates, short life cycle and relatively easy methods of sampling, provide statistically significant sample size [10] as well as fewer chances of diminishing the population.

Habitat destruction and fragmentation, abiotic and biotic environmental changes, as well as oil spills and other pollutants are some of the factors deteriorating the health of the saltmarshes in coastal Louisiana [3,11–13]. In recent years, coastal Louisiana saltmarshes have been significantly affected by the release of crude oil via numerous oil seeps, leaks, and spills, with the most recent major event being the *Deepwater Horizon* (DWH) oil spill in 2010 which released ~4.6 million barrels of crude oil into the northern Gulf of Mexico (nGOM) [14]. While much of the oil and weathered oil residues remained offshore and were subsequently acted upon by several weathering processes, a significant amount of weathered oil residues came ashore, impacting coastal Louisiana marshes and beaches [14,15–19]. Coastal Louisiana represents 65% of total nGOM oiled shoreline and 95% of total oiled marshes after the DWH oil spill [16,20]. Weathered oil residues along coastal Louisiana had a very heterogeneous distribution on a scale of “trace” to “heavily oiled” and were either stranded or buried along the impacted shorelines as determined by the Shoreline Cleanup and Assessment Technique (SCAT) [17, 21,22]. Organisms, including terrestrial arthropods, residing in these coastal areas may have been affected directly, by the exposure to toxic components in oil residues, or indirectly, by the reduced availability of key food sources, such as plants and amphipods.

Several tropical weather events, including Tropical Storm Lee in 2011 and Hurricane Isaac in 2012, crossed the initial DWH-impacted coastal areas of Louisiana and resulted in a re-mobilization of oil residues from the initially oiled marshes [23]. These storm events either transported the oil residues farther into the marshes in a very non-homogenous manner, or they buried oil residues more deeply within associated soil pore space and animal burrows [23]. The re-mobilization of oil residues during storm events is a significant environmental concern for Louisiana’s coastal saltmarshes [15,23–26]. Re-mobilization of buried oil can cause chronic exposure of saltmarsh organisms, including terrestrial arthropods, which can adversely affect their abundances and community compositions [23,27]. Arthropods have a main role in food webs, which affect ecosystem functions as arthropods inhabit high diversity of micro-habitats and niches [10,28]. The Exxon Valdez oil spill in Prince William Sound had significant long-term negative impacts on the saltmarsh birds, intertidal arthropods and their habitat [29]. Thus, it is imperative to study the impacts of such oiling and re-oiling events on arthropod communities, as a proxy, to understand the overall health of these saltmarsh ecosystems.

We aim to use saltmarsh arthropods as indicators in determining the impacts of DWH oil spill (Macondo Oil) through re-oiling/redistribution by Hurricane Isaac on terrestrial arthropods in Louisiana saltmarshes by comparing abundances and community composition at oiled and reference sites. Despite a large amount of research being conducted in this region, few studies have focused on the abundance and distribution of selected arthropods in coastal Louisiana [30,31]. To the best of our knowledge, none of this previous researches has studied the coupled effects of the DWH oil spill and subsequent hurricanes on the overall arthropod communities. We hypothesized that there would be differences in the terrestrial arthropod communities among the reference, lightly-oiled, and heavily-oiled sites in Louisiana saltmarshes. This study will provide an outlook on the trophic effects of oil pollution on saltmarsh ecosystems.

## Materials and methods

### Study area

**Fig 1:**
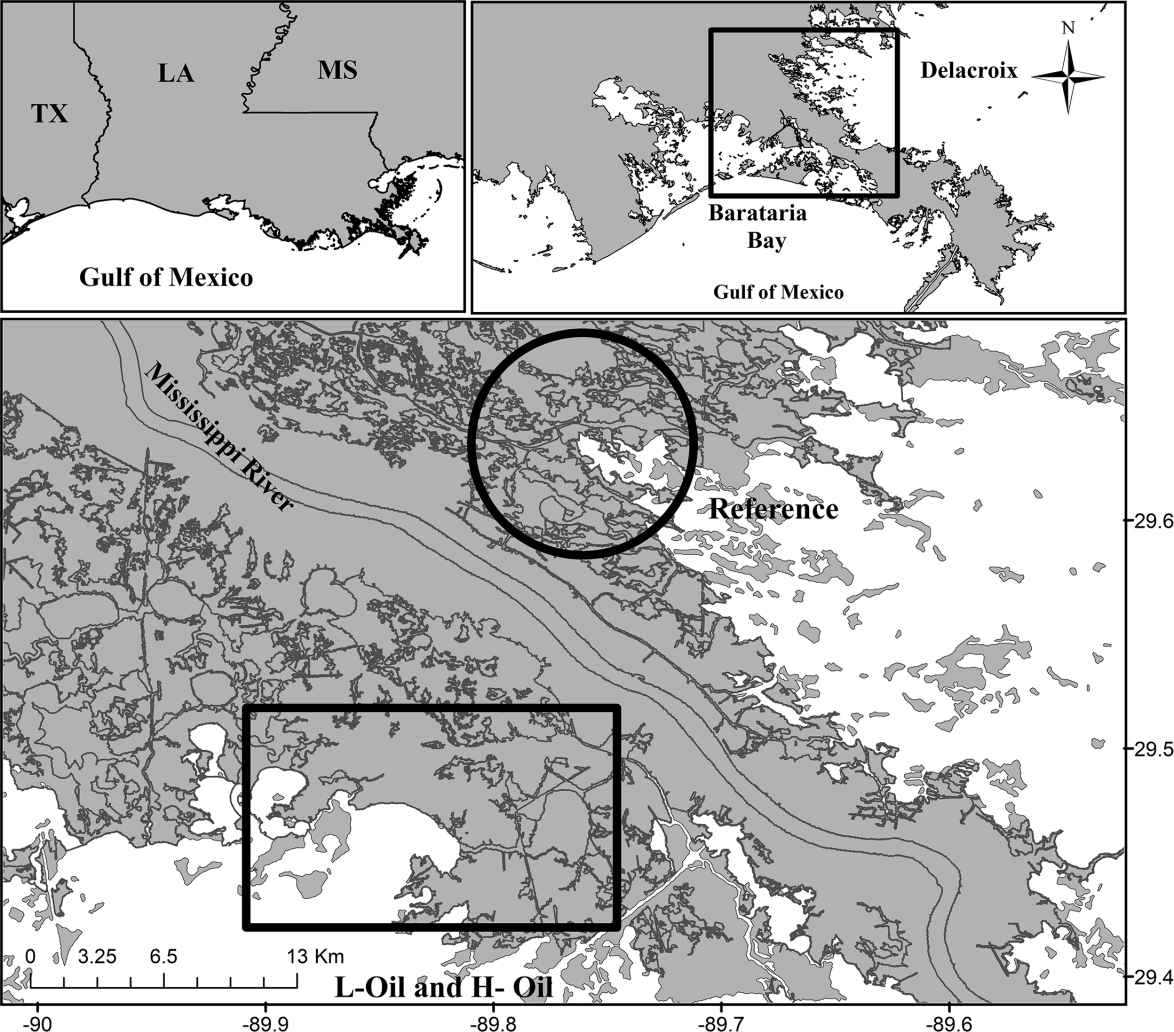
Sampling locations in Barataria Bay, lightly-oiled (L-Oil) and heavily-oiled (H-Oil) sites (Rectangle) and Delacroix reference sites (Circle) in Louisiana.

Study sites were located in Barataria Bay and Delacroix, Louisiana (Fig 1 and Table 1). Both study regions are characterized by low tidal ranges and have saltmarsh vegetation dominated by *Spartina alterniflora*, *Juncus roemearianus*, *Distichlis spicata*, and *Avicennia germinans* [32]. Seven study sites were selected in Barataria Bay, three of which were classified as lightly-oiled (L-Oiled) and four of which were classified as heavily-oiled (H-Oiled) with DWH residues. The heavily-oiled sites were classified based on the initial DWH oiling, which was determined using polycyclic aromatic hydrocarbon (PAH) concentrations reported by the National Oceanic and Atmospheric Administration (NOAA) Natural Resource Damage Assessment (NRDA) Workplans and Datasheet for shoreline oil levels in 2010 and 2011 (http://www.gulfspillrestoration.noaa.gov/oil-spill/gulf-spill-data/) (Fig 1 and S1 Fig). The NOAA-NRDA and the Coastal Waters Consortium (CWC) PAH data showed no visible oil or low PAHs (<200 ng/g of sediment) in the lightly-oiled sites and higher PAHs (>200 ng/g of sediment). However, sediments collected in November and December of 2012 at these same sites showed higher PAH concentrations (>200 ng/g), suggesting circulation, transportation, and oiling in new locations which were not initially oiled by the DWH residues. This redistribution of DWH oil residues occurred primarily during a number of tropical storms passing through the area, Hurricane Isaac in 2012 being the most significant among them [23]. The NOAA-NRDA Workplans and Datasheet for PAHs reported no DWH oil in Delacroix in 2010 and 2011, and low PAHs (<200 ng/g of sediment) in 2012 and 2013. Forensic biomarker (hopene, steranes and steroids) based oil finger printing showed that the Delacroix sites were not contaminated by MC252 (DWH oil). Therefore the observed PAHs concentration (<200 ng/g of sediment) were either background PAHs or contaminated by some other sources. Accordingly, Delacroix study sites were considered reference sites in the present study (S1 Fig). Several independent CWC studies have used these sites and similar classification [33,34]. CWC-I and CWC-II research collaborators, funded by the Gulf of Mexico Research Initiative (GoMRI), have collected time series sediment samples (top 5 cm) from Barataria Bay and Delacroix sites and have analyzed them for total PAHs as an indicator of the impacts of the DWH oil residue in these saltmarshes. PAH analysis is not the primary focus of the present study. However, a brief description of PAH analyses and surface sediment PAH distributions relevant to the study may be found in the Supplementary document (S2 File, S2 and S3 Figs). The details on the sample collection, extraction, and analysis for PAHs using GC/MS have been previously published by Turner et al. [27] and Adhikari et al. [35,36]. The results of the time-series surface sediment PAH analyses (S2 and S3 Figs) align very well with previously reported results [23], and further indicate the re-distribution of DWH oil residues by the Hurricane Isaac.

**Table 1:** Geographic coordinates for the treatment sites: heavily oiled, lightly oiled and reference sites.

Sites	Latitude	Longitude
Heavily Oiled (A)	29.45674	-89.88654
Heavily Oiled (B)	29.47644	-89.84792
Heavily Oiled (C)	29.46861	-89.86333
Heavily Oiled (D)	29.47295	-89.83218
Lightly Oiled (A)	29.45298	-89.77909
Lightly Oiled (B)	29.46007	-89.87556
Lightly Oiled (C)	29.46880	-89.75734
Reference (A)	29.60802	-89.78548
Reference (B)	29.62381	-89.78520
Reference (C)	29.64561	-89.80005

### Ethics statement

Insects and spiders from 2013 and 2014 were collected under Louisiana Department of Wildlife and Fisheries (LDWF) scientific collecting permit statewide LNHP-12-047, WL-Research-2012-03 and LNHP-14-039. This study was carried out in strict accordance with the recommendations in the Guide for the Care and Use of Laboratory Animals of the National Institutes of Health. Protocols were approved by the Institutional Animal Care and Use Committee of the, LSU AgCenter (IACUC A2012-05). The field study did not involve endangered or protected species.

### Sample collection

We collected terrestrial arthropods (insects and spiders) from April to June in 2013 and 2014 in the saltmarsh vegetation. We used sweep nets along linear transects, measured from the edge of the marsh to 20m inland (40m x 2m plots). The sweep net is one of the most effective and commonly used methods for sampling terrestrial arthropods [37–43] however sweep net method does not account for arthropods living inside plant stems and ground-dwelling arthropods. We used a 15-inch diameter standard sweep net and collected samples by swinging the net from side to side in a 180° arc (S4 Fig). We sampled once a month from each site, in the morning (between 6:30 am and 12:00 pm). We sampled in lightly-oiled and heavily-oiled sites on the same day, and in reference sites on the following day. Rainy days were excluded to avoid the sampling biases. We transferred arthropods from nets into plastic ziploc bags containing 95% ethanol for transport to the lab. We then sorted the samples and classified taxa to order and family using appropriate taxonomic keys and the morphospecies approach [44–46] and then the numbers were counted and recorded. All spiders (Order Araneae) were classified as one group for all calculations and statistical analyses.

### Statistical analysis

We performed Shapiro-Wilk test to determine normality of the datasheet and the test showed distribution was highly skewed, thus, the data were log transformed to normalize. Convergence criteria was met for all individual arthropods during statistical analysis. We used a general linear model (PROC MIXED in SAS) to compare the effects of treatment (levels of oil impacts—references, lightly-oiled sites, and heavily-oiled), year (2013 and 2014), and the treatment-by-year interaction on the abundance of arthropods. We assumed that the taxonomic richness, abundance of arthropods and seasonal changes over three months (April to June) spring/summer was minimal. Hence, we considered the three-month sample collection as replicated and the mean of the samples were used in the diversity index. We performed posthoc comparison (Tukey’s HSD p<0.05) to determine significant effect. We used a two way ANOVA to compare the arthropods diversity measure index (Shannon and Weaver Index) and taxonomic richness (Menhinick’s species richness index) among treatments and years. We used Principal Component Analysis (PCA) to visualize arthropods community structure for all the treatment and year for which the monthly data were lumped together. All the statistical analyses were performed using SAS 9.4 software [47] and a P-value of 0.05 was used to determine the level of significance for the comparisons.

## Results

### Arthropod data

**Fig 2:**
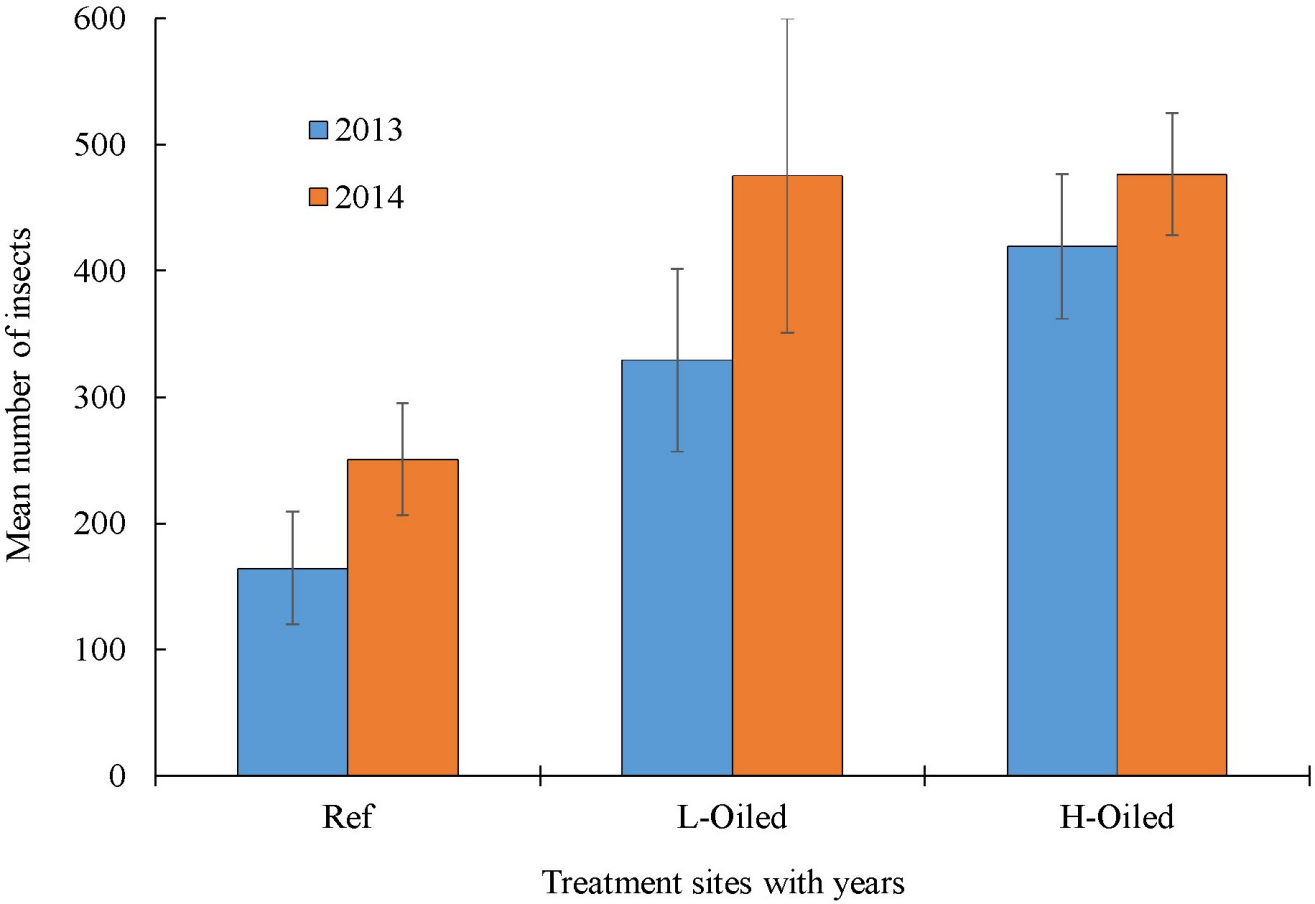
Mean (±SE) number of insects sorted per sweep net sample in all treatment sites (references, light oiled and heavily oiled sites) in 2013 (blue) and 2014 (orange).

We collected a total of 21,732 arthropods, out of which 9,476 were collected in 2013 and 12,256 in 2014. The mean number (± SE) of terrestrial arthropods collected in 2013 was 165 ± 134 in reference sites, 329 ± 217 in lightly-oiled, and 420 ± 199 in heavily-oiled (Fig 2). In 2014, we collected 251 ± 133 arthropods in reference sties, 476 ± 374 in lightly-oiled, and 477 ± 167 in heavily-oiled (Fig 2). The number of total arthropods varied significantly (P<0.05) among the treatment sites in 2013, with heavily-oiled sites having significantly more arthropods followed by lightly-oiled and reference sites (Fig 2). In 2014, the total arthropods in reference sites was again lower than the totals in heavily-oiled and lightly-oiled sites, which did not significantly differ from each other.

### Treatment effects by taxon

**Fig 3:**
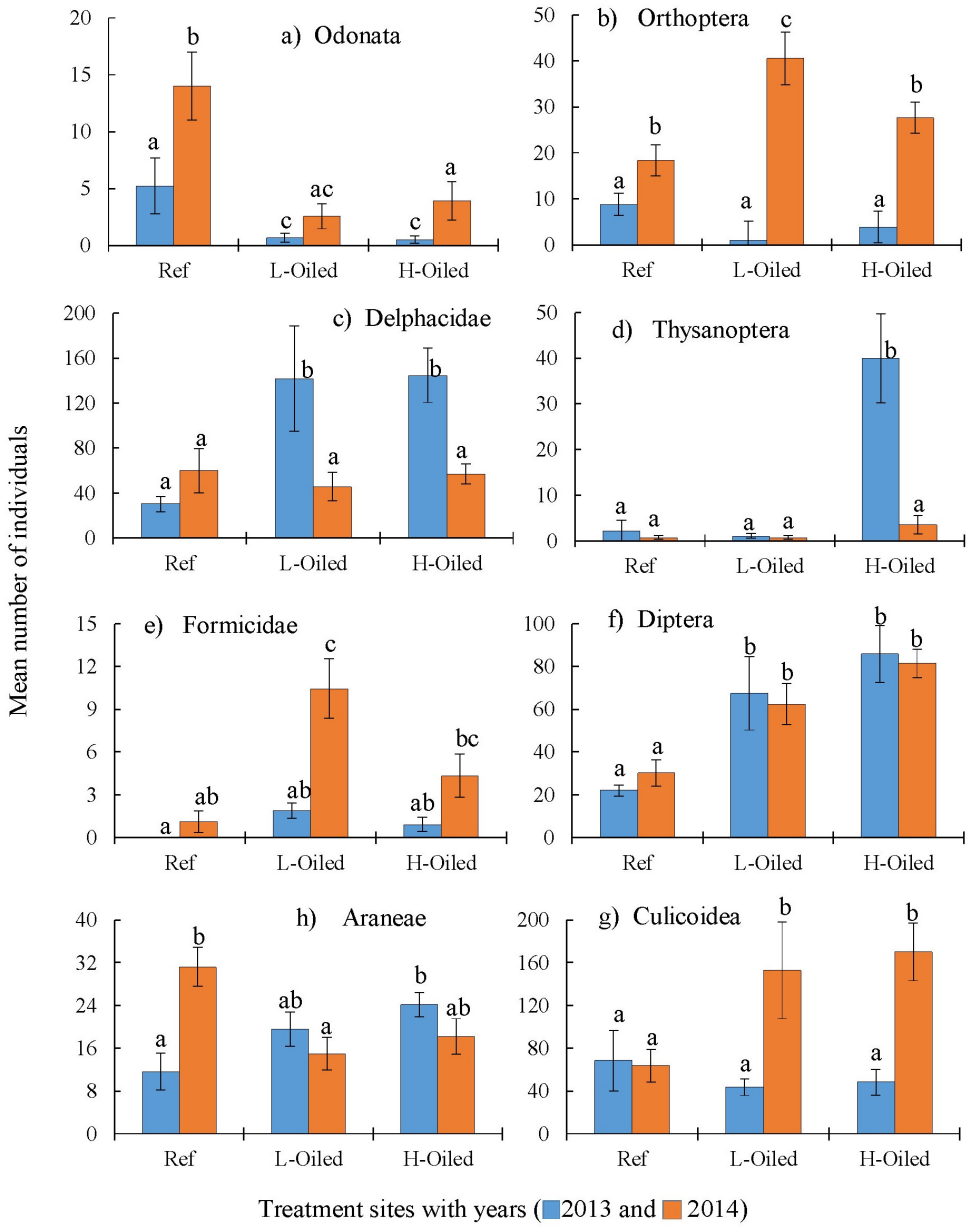
Mean (±SE) abundance of individual group of arthropods collected per sweep net sample in reference, light oiled and heavily oiled sites in 2013 (blue) and 2014 (orange).

The effects of treatment and sampling year are not the same for all the arthropod groups collected in this study. Some of the groups show significant interaction (treatment-by-year) effects, while others show only significant main effects, i.e., either among treatments, years, or both (Table 2). Here we present the results for eight selected group of arthropods (Fig 3). The results for the rest of the arthropod groups are included in the supplementary documents (S2 File and S5 Fig). The degree of freedom for treatment (df = 7), year (df = 47) and treatment*year interaction (df = 47) is same for all the arthropods.

**Table 2:** Effect of treatment (heavily oil, lightly oiled, and reference), year (2013 and 2014) and their interaction (treatment*year) on the insects and spider (p values). The degree of freedom for treatment (df = 7), year (df = 47) and treatment*year interaction (df = 47) is same for all the arthropods.

Arthropods	F value			p value		
	Treatment	Year	Interaction (Treatment*Year)	Treatment	Year	Interaction (Treatment*Year)
Odonata	10.72	14.86	1.51	0.00	0.00	0.23
Orthoptera	0.01	78.00	3.61	0.99	0.00	0.03
Miridae	17.25	0.82	0.02	0.00	0.35	0.98
Delphacidae	3.54	1.02	1.91	0.04	0.32	0.16
Blissidae	8.69	6.49	1.39	0.00	0.02	0.26
Thysanoptera	5.98	2.82	2.74	0.01	0.11	0.07
Coleoptera	6.70	3.50	4.26	0.00	0.08	0.02
Formicidae	4.64	5.07	0.56	0.01	0.04	0.55
Pompiloidea	3.50	13.26	1.13	0.03	0.00	0.33
Lepidoptera	0.08	1.86	1.26	0.94	0.18	0.29
Diptera	18.24	0.06	0.06	0.00	0.80	0.94
Culicoidea	1.37	6.65	1.32	0.26	0.02	0.27
Araneae	0.90	2.16	8.84	0.41	0.16	0.00

#### Odonata

The order Odonata comprises the dragonflies and damselflies, and both were present in samples collected throughout the sampling period. Odonatans were significantly more numerous (P = 0.04) in the reference sites than in lightly-oiled or heavily-oiled sites in both years (Fig 3a and Table 2). Dragonflies and damselflies may have had lower numbers on oiled sites because of decreased prey availability.

#### Orthoptera

Ground-dwelling, omnivorous crickets from the order Orthoptera showed a significant treatment-by-year interaction (P<0.05), and were significantly more abundant in all study sites in 2014 compared to 2013 (Fig 3b and Table 2). Crickets are interesting, because some of members of their group live in close contact with the sediment. Even though they feed on plants and can feed on dead material, they are in direct contact with the contaminated soil. Herbivorous Orthopteran, long-faced katydids, populations increased in oiled areas possibly indicating an increase in plant stress. Orthoptera were clearly affected by oil spill and Hurricane Isaac and made a resurgence across the ecosystem in 2014.

#### Delphacidae

Planthoppers (family Delphacidae) were one of the most abundant groups of arthropods collected. In 2013, reference sites had a significantly lower (P<0.01) number of Delphacids than lightly-oiled and heavily-oiled sites. However, the Delphacid abundances were similar across all sites in 2014 (Fig 3c and Table 2). Heavily-oiled and lightly-oiled sites experienced significant decreases (P<0.05) in the population of Delphacidae in 2014 compared to 2013, but the Delphacidae population increased in 2014 in reference sites although it was not significant (P = 0.1) then 2013. In 2013, Delphacids were in higher number in oiled sites than in 2014, which was likely due to the immediate combined stress of oil and Hurricane Isaac. Delphacidae were similar across all sites in 2014, suggesting the population of Delphacidae, as a whole, might be back to normal.

#### Thysanoptera

Thrips (order Thysanoptera) were significantly more abundant (P<0.01) in 2013 at heavily-oiled sites compared to all other sites and years (Fig 3d and Table 2). Thrips are herbivores and can increase herbivory in response to an increase in plant stress [48,49]. Thus, the high number of Thrips in heavily-oiled sites in 2013 may be attributable to the stress experienced by saltmarsh plants after Hurricane Isaac and the re-distribution of DWH oil residues.

#### Formicidae

There was a significant difference in the number of ants (family Formicidae) across treatments (P = 0.02) and years (P<0.01) (Fig 3e, Table 2). All treatment sites had significantly more (P = 0.02) ants in 2014 than in 2013. Lightly-oiled sites in 2014 had a significantly higher number of ants than all other sites and years. Ants were absent in the samples collected in reference sites in 2013. Ants were greatly affected by Hurricane Isaac, and we observed the destruction of ant colonies in the field. However, our data show ants present in sweeps from all areas in 2014 presumably because of mating flights that occurred in April.

#### Diptera

Dipteran flies include the families Ulidiidae and Chloropidae. Flies were significantly lower (P<0.01) in reference sites than in lightly-oiled and heavily-oiled sites in both years (Fig 3f and Table 2). However, within treatments there were no significant differences in fly abundance between 2013 and 2014 (Fig 3f and Table 2). Recently, Husseneder et al. [31] reported that Dipterans were negatively impacted by oil. We did not find that heavily-impacted sites had fewer flies, possibly due to an abundance of dead and decaying material at the oiled sites and because flies are highly mobile, able to colonize compromised habitats quickly.

#### Culicoidea

The midges and mosquitoes (super family Culicoidea) were grouped and counted together. In 2013, the numbers of Culicoideans did not significantly differ among treatment sites (Fig 3g and Table 2). The Culicoidean population was significantly higher (P<0.01) in heavily-oiled and lightly-oiled sites in 2014 than in 2013 (Table 1), but it was not significantly different (P>0.20) between the two years in reference sites. Culicoideans, which filter-feed on detritus as larvae and are predatory as adults, had an increased population in oiled sites in 2014 than in 2013 but reference sites had similar numbers in both years.

#### Araneae

Spiders (order Araneae) were significantly fewer in reference sites in 2013 than in lightly-oiled and heavily-oiled sites. However, their abundance in reference sites significantly increased in 2014 than in 2013 (P<0.01). Their abundance in the reference site was higher than in heavily-oiled and lightly-oiled sites (Fig 3h and Table 2). Little is known about how spiders in the saltmarsh respond to stress. However, it is possible that the increase in population in 2014 was related to a recovery of their prey populations.

### Taxonomic richness and diversity metrics

**Fig 4:**
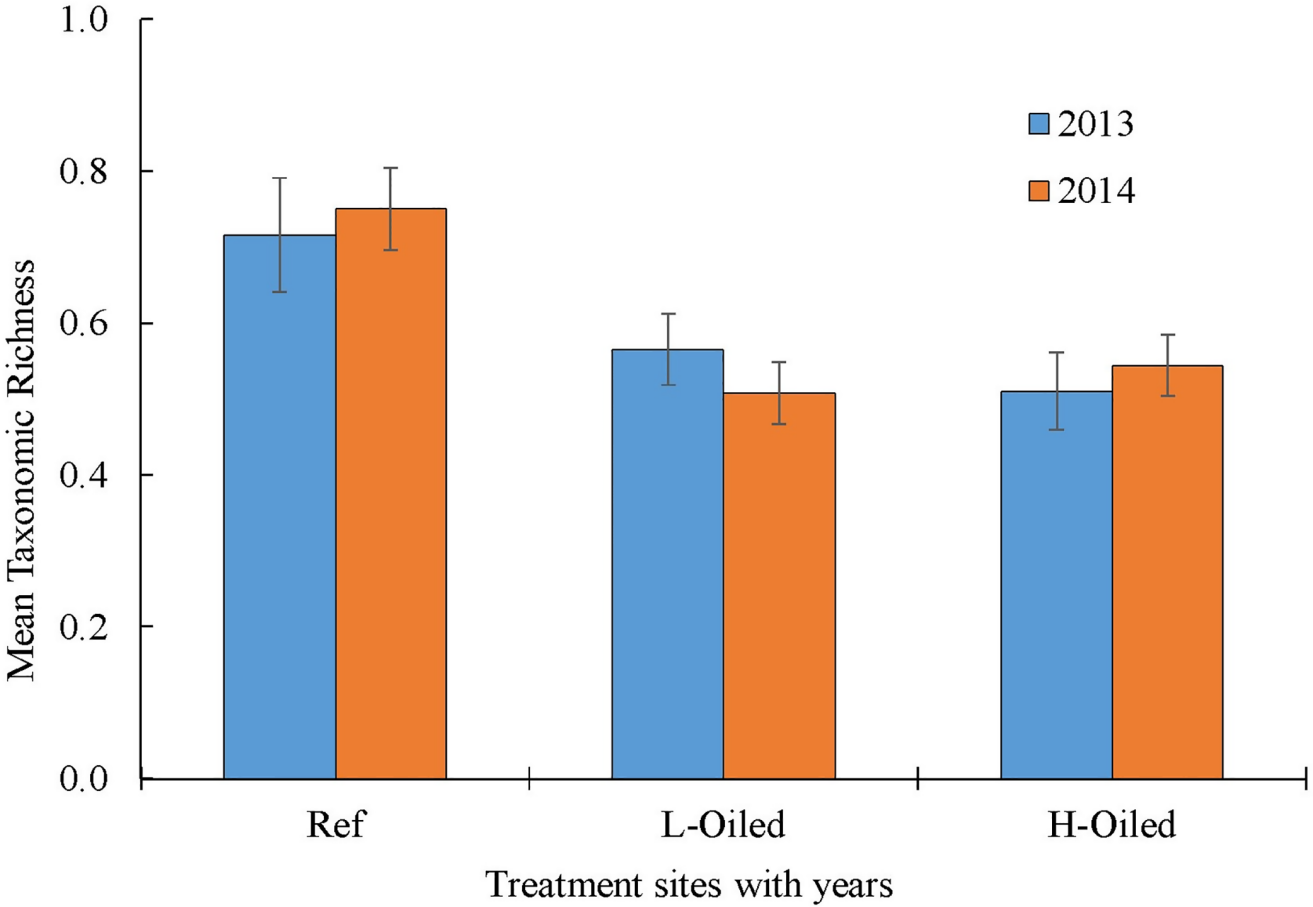
The mean taxonomic richness across the treatment sites and years (references, light oiled and heavily oiled sites) in 2013 (blue) and 2014 (orange).

**Fig 5:**
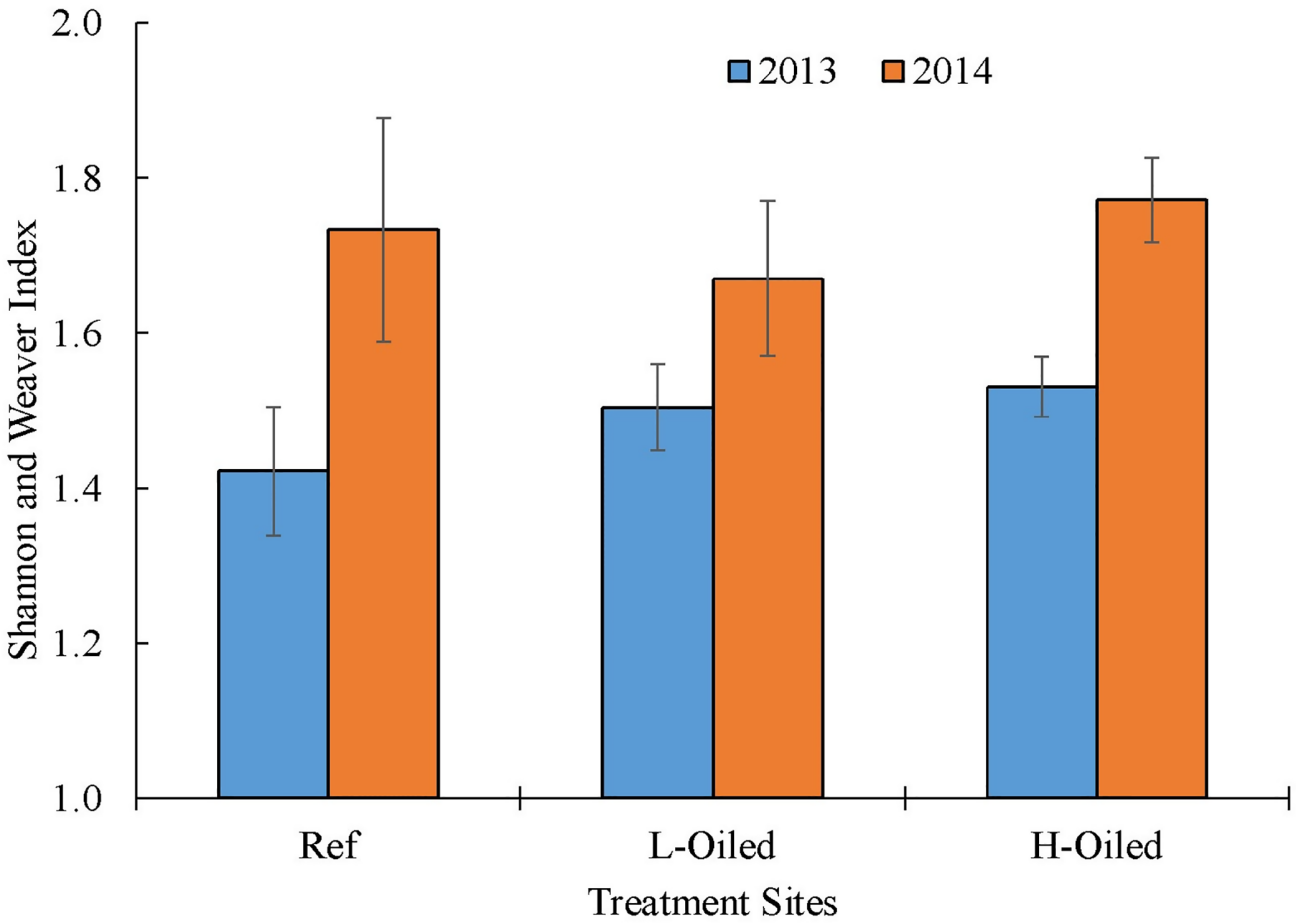
Mean ((±SE) diversity measurement in terms of Shannon and Weaver Index for treatments (reference, lightly oiled and heavily oiled) in 2013 (blue) and 2014 (orange).

A total of 28 families of insects were collected throughout the sampling period. Some of these families were represented by a single or few individuals and were thus combined to the level of order. A condensed total of 15 families and orders were used for taxonomic richness and diversity calculations. We calculated the taxonomic richness using Menhinick’s species richness index (D_Mn_) and the diversity using the Shannon and Weaver Diversity Index (Figs 4 and 5). The references sites had significantly higher (P<0.05) taxonomic richness both in 2013 and in 2014 than the lightly-oiled and heavily-oiled sites (Fig 4). However, the taxonomic richness did not significantly differ between the lightly-oiled and heavily-oiled sites in both sampling years. All sites across treatments had significantly higher mean diversity in 2014 compared to 2013 (P<0.05; Fig 5). Overall, the diversity index showed the temporal variation on diversity for all the sites whereas no spatial variation was observed (Fig 5).

### Community structure

**Fig 6:**
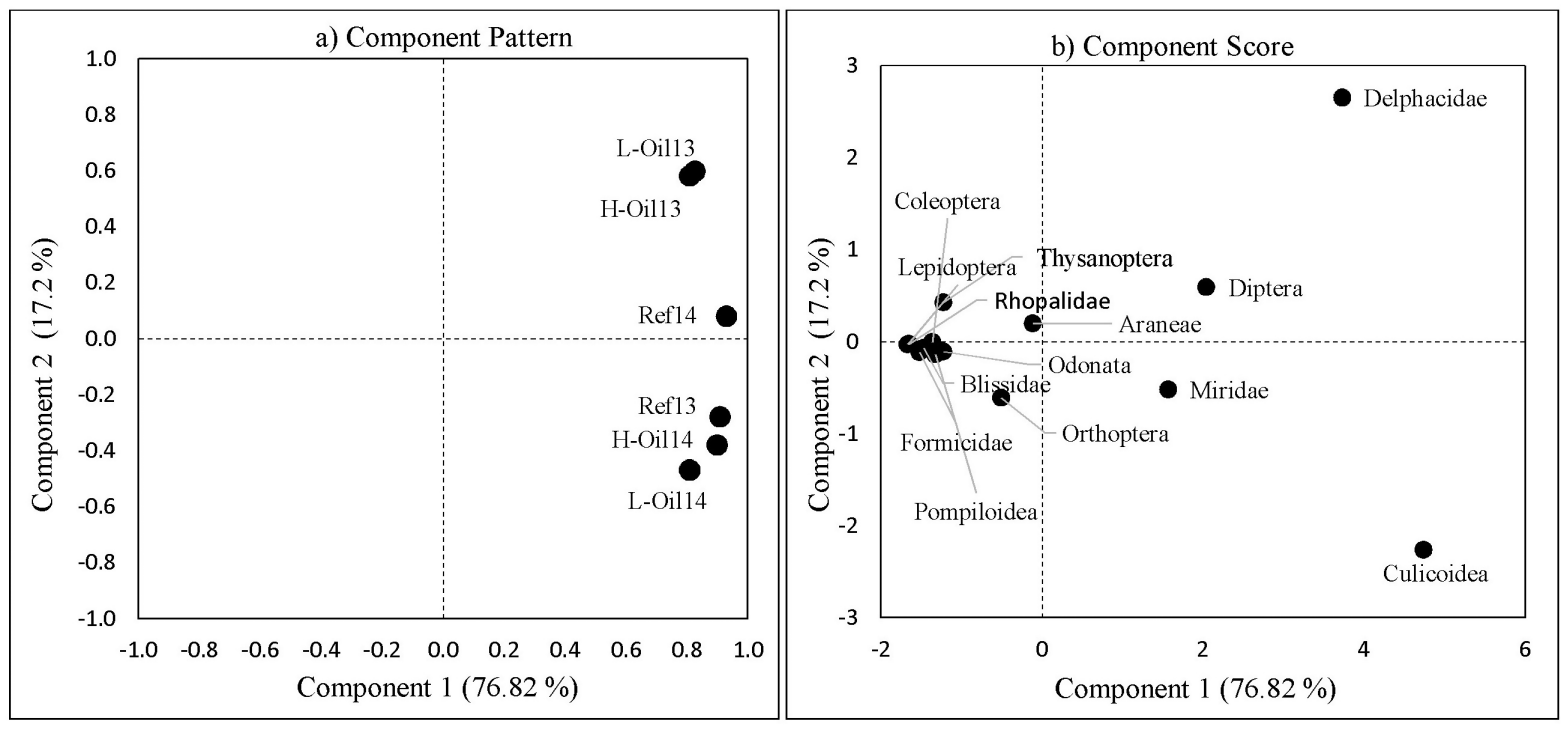
Principal Component Analysis (PCA); a) Community structure analysis and (b) Component score for each individual family/order for the treatment sites and year.

**Fig 7:**
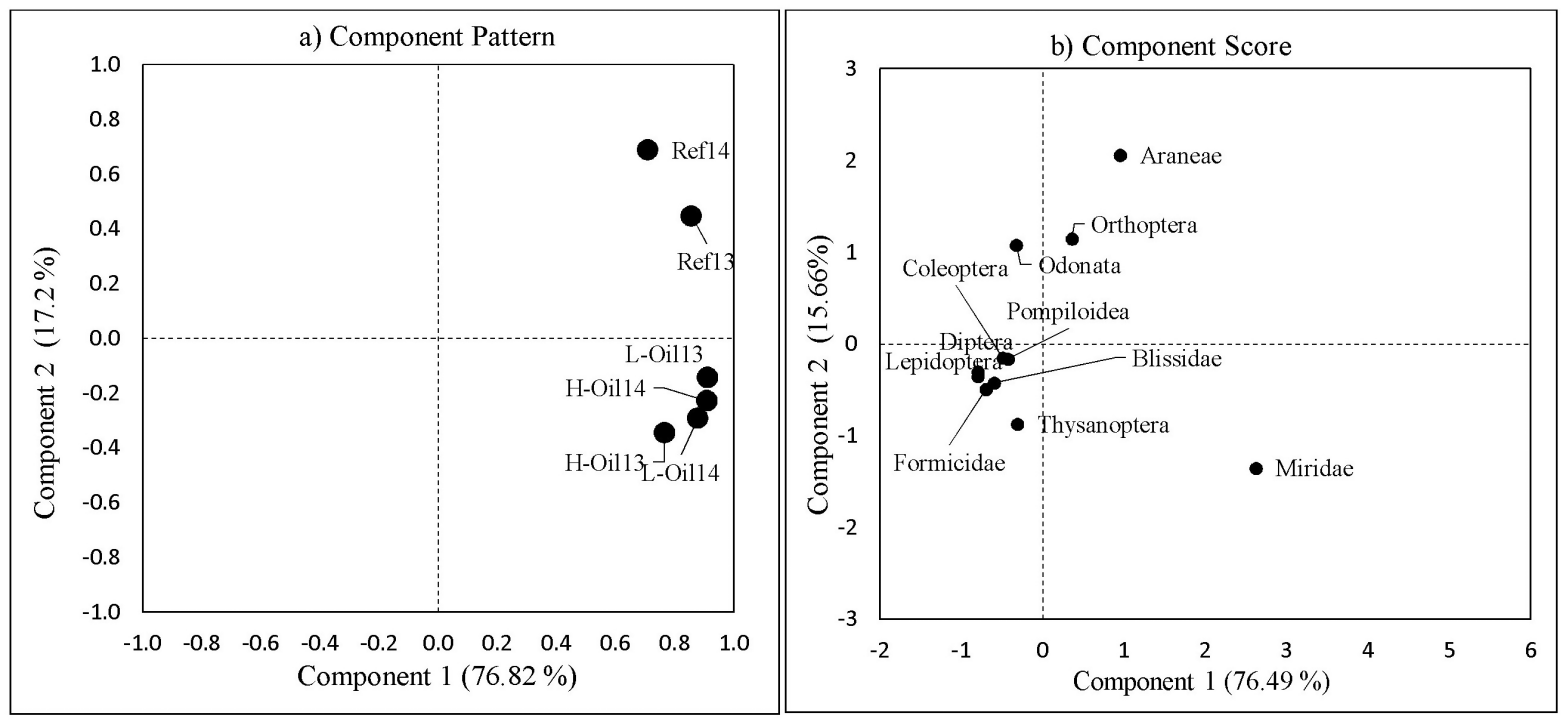
Community structure analysis using Principal Component Analysis II excluding Delphacidae and Culicoidea (PCA II) a) Community structure analysis and (b) Component score for each individual family/order for the treatment sites and year.

The first two principal components (PCs) explained 94% of the variation (76.82% by PC1 and 17.2% by PC2) of the insects community structure (Fig 6). None of the sites clustered by treatment (Fig 6a). However, the lightly-oiled and heavily-oiled sites in 2013 clustered very closely together. The 2014 lightly-oiled and heavily-oiled sites were similar to each other and to the 2013 reference sites. Most of the insects were grouped together along the PC1 component score (Fig 6b). The high abundances of the Delphacidae and Culicoidea in 2013 for oiled sites mainly influenced the community structure among sites. In order to understand how the Delphacidae and Culicoidea influenced the community structure, we re-ran the PCA (PCA II) without Delphacidae and Culicoidea (Fig 7a and 7b). After removing the Delphacidae and Culicoidea, the community structures of reference sites was clearly distinct from the community structures of heavily-oiled and lightly-oiled sites, which clustered together (Fig 7).

## Discussion

### Arthropod communities

Oil pollution and Hurricane Isaac had various effects on different terrestrial arthropods in our study. Each group of arthropods measured responded differently over treatments, years, and treatment-by-year interactions. Some of the arthropod groups were affected in years three (2013) and four (2014) after the DWH spill, and some apparently were not. This indicates that certain groups of arthropods may serve as better indicators of marsh stress than others. After initial recovery from Hurricane Isaac, Odonata and Araneae were significantly higher in reference sites than lightly-oiled and heavily-oiled sites. It should be noted that this study began several months after Hurricane Isaac spent ~72 hours over south Louisiana, flooding the marsh with storm surge. Hurricane Isaac passed through the study sites from August 29 to September 3, 2012 [50], disturbing the ecosystem and redistributing DWH oil residues [23], as indicated by higher total sediment PAH concentrations (S2 Fig). Our sampling began during the first reproductive season for most insects following Hurricane Isaac, and we assume our data represent any effects of oil re-distribution and hurricane disturbance. Our results suggest that terrestrial arthropod groups respond differentially to re-oiling. McCall and Pennings [30] found that all saltmarsh invertebrates were strongly affected suppressed by 50% by oil contamination within the first year following DWH (2010–2011). This may be due to the timing of both studies. McCall and Pennings [30] showed that arthropod population in oiled sites recovered within the first year and were similar to the control sites.

It has been hypothesized that after disturbance in multi-trophic food webs, predators, such as wasps and spiders, will increase over time as the result of increases in herbivores [51]. Outbreaks of herbivorous insects (e.g., Orthoptera, Thysanoptera) are associated with various environmental stressors or disturbances [52,53]. An outbreak of herbivores may be promoted by various factors, including plant stress [49], absence of top-down control by parasitic wasps [54], shifts in predator/parasitoid feeding preferences [55], and an overall reduction in predation [56]. Herbivores Miridae abundance in lightly-oiled sites (S5a Fig) may potentially be linked to stress in plants due to higher amounts of nitrogen [57]. Predators may in turn alter the abundance of herbivores in the ecosystem [58]. Spiller and Schoenor [59] explained that predators, such as web spiders, may regulate herbivore abundance through direct predation (population decrease) or by feeding on predators/parasitoids that feed on herbivores (population increase). The increase in predators such as Araneae would reduce the abundance of herbivores such as Delphacidae [60]. The results from our study are consistent with direct effects of predation [60], as there was a decrease in Delphacidae and an increase in Araneae in reference sites.

All study sites were disturbed by Hurricane Isaac with high seawater storm surge, wind and precipitation. The reference sites had lower abundances of herbivorous insects in 2013 than in 2014, while the opposite trend was observed in lightly-oiled and heavily-oiled sites. This may be evidence for the plant stress hypothesis. Overall, herbivore abundance was greater than omnivore and predator abundances, which partially supports the trophic distribution pyramid described by Speight et al. [1], Pearson and Dyer [61] and Turney and Buddle [62] that more than 50% of arthropods in the ecosystem are herbivores. The significant increase of Araneae (predators) in reference sites in 2014 may be one of the reasons for observed decrease in herbivores and omnivores, which was not observed in lightly-oiled and heavily-oiled sites.

### Taxonomic richness and diversity metrics

Taxonomic richness is one of the simplest measures to represent the number of species present in certain area [63] and with two basic components: number of species (family), and evenness of distribution [64,65]. Shannon’s diversity index measures the species diversity in an ecosystem and provides collective information about the community composition than simply richness. It was observed that the overall abundance of terrestrial arthropods was significantly higher in 2014 than in 2013 for all sites (Fig 2). Diversity also increased in 2014 versus 2013 across all sites, which provides evidence of whole-ecosystem recovery within 6–18 months after Hurricane Isaac. The diversity increase may also represent part of a multi-year recovery of insect and spider populations after DWH. Possible reasons for higher individual arthropod numbers in are: (1) groups that are expert fliers and colonizers were more abundant in 2014 than in 2013, (2) groups that feed on detritus or stressed plants were more abundant in oiled sites than reference sites. The abundance and diversity of terrestrial arthropods is influenced by immigration from adjacent habitats [66]. Terrestrial arthropod communities can experience seasonal changes in trophic levels depending upon the availability of resources, such as food, vegetation coverage [63,67]. Taxonomic richness was higher at nearly 0.8 in reference sites in both years than in lightly-oiled and heavily-oiled sites, where it ranged from 0.5 to 0.6 index. This suggests that even after four years, taxonomic richness may still be negatively affected by presence of DWH oil residues. Magurran [65] suggested that species richness could be regarded as good indicator of the health of an ecosystem and is often used to conduct the environmental assessment studies [68]. The higher taxonomic richness for both sampling years in the reference sites in the present study suggests reference sites may be healthier compared to the lightly oiled and heavily oiled sites.

### Conclusions

Our results suggest that both DWH oil residues and Hurricane Isaac may have directly and indirectly impacted the terrestrial arthropods of the Louisiana saltmarsh. However, the degree of impact varied among taxa. In contrast to previously reported results [30], we are still observing the impacts of DWH oiling more than four years after the 2010 spill. The long-term impacts of oiling and re-distribution during tropical storm events on terrestrial arthropods should not be overlooked. Terrestrial arthropod communities worldwide are capable of recovery after environmental and ecological disturbance, though the rate of recovery for specific arthropods may differ in each location. Baseline and long-term data, not just on the coast of the Gulf of Mexico, but are needed in many ecosystems to quantify and explain the effects of oil spills and other pollutants on recovery and succession of marsh ecosystems. Hurricanes form an important part of the oil contamination story, as they can potentially remobilize and redistribute oil residues in coastal ecosystems. Terrestrial arthropods, with their short lifecycles and generation times, may serve as ideal indicators for monitoring the impacts of these ecosystem disruptions both for short-term and long-term ecosystem recovery.

## Supporting information

S1 File (DOCX)

S2 File (DOCX)

S1 Fig Oil observation on ground NOAA/NRDA data 2010/2014 in Louisiana coast (Source: NOAA/NRDA 2015, http://www.gulfspillrestoration.noaa.gov/oil-spill/gulf-spill-data/). (TIFF)

S2 Fig The average concentrations of total PAHs (ng/g) in Barataria Bay sediments impacted by the *Deepwater Horizon* oil spill. (TIFF)

S3 Fig The average concentrations of total PAHs (ng/g) in reference sites (Delacroix) sediments. (TIFF)

S4 Fig Field sampling of terrestrial arthropods using sweep net in Barataria Bay in 2014. (TIFF)

S5 Fig Mean (±SE) abundance of individual group of arthropods collected per sweep net sample in reference, light oiled and heavily oiled sites in 2013(blue) and 2014 (orange). (TIFF)
