# Correction: Coupled effects of oil spill and hurricane on saltmarsh terrestrial arthropods

**DOI:** 10.1371/journal.pone.0199467

**Published:** 2018-06-15

**Authors:** Wokil Bam, Linda M. Hooper-Bui, Rachel M. Strecker, Puspa L. Adhikari, Edward B. Overton

The following information is missing from the Data Availability and Acknowledgements sections: This research was made possible by a grant from The Gulf of Mexico Research Initiative. Data are also publicly available through the Gulf of Mexico Research Initiative Information & Data Cooperative at https://data.gulfresearchinitiative.org (GRIIDC, doi: 10.7266/N7K935FT).
