# Supplementary material for: Coupled effects of oil spill and hurricane on saltmarsh terrestrial arthropods
Source: PLoS One. 2018 Apr 11;13(4):e0194941. doi: 10.1371/journal.pone.0194941 (PMC5895010; doi:10.1371/journal.pone.0194941)
Supplement: S1 File — (DOCX) [file pone.0194941.s001.docx]

**S1. Methods**

***Chemical Analysis and Oil Redistribution***

As part of the Coastal Waters Consortium (CWC-I and CWC-II) study into impacts from the DWH oil spill, samples of the top 5 cm of surface sediment were collected by the authors (WB and LMHB) at sites from Turner et al. [15,27] adjacent to the shoreline from plots in Barataria Bay and Delacroix. These were analyzed for total polycyclic aromatic hydrocarbons (PAHs). The details on the sample collection, extraction, and analysis for PAHs using GC/MS have been previously published by Turner et al. [27] and Adhikari et al. [35,36]. The surface sediment samples (n=850) showed average concentrations of total PAHs from 260 to 940 ng/g (S2 Fig). Detailed study on PAHs analysis is out of the scope of this study. However, the sediment PAH analysis provided us very important information on the re-distribution of buried oil in coastal marshes following Hurricane Isaac. There are no significant differences in the concentrations of total PAHs in sediment samples (n=170) from 2011 and in samples (n=106) from 2012 sediment (S2 Fig). However, the concentrations of total PAHs in the marsh sediments collected in 2013 were significantly higher than in other years (179 sediment samples in 2013; 311 sediment samples in 2014; and, 84 samples in 2015) (S2 Fig), which may be due to the redistribution of DWH oil by tropical storms and Hurricane Isaac (September of 2012). The concentration of total PAHs decreased significantly in 2014 and 2015 compared to 2013 (S2 Fig). The PAHs concentration in samples in reference sites ranged between 60-200 ng/g (n = 125) (S3 Fig).
