# Supplementary material for: Coupled effects of oil spill and hurricane on saltmarsh terrestrial arthropods
Source: PLoS One. 2018 Apr 11;13(4):e0194941. doi: 10.1371/journal.pone.0194941 (PMC5895010; doi:10.1371/journal.pone.0194941)
Supplement: S2 File — (DOCX) [file pone.0194941.s002.docx]

**S2. Results**

**S2.1 Miridae**

Miridae were more abundant in lightly-oiled sites in 2014 compared with lightly-oiled in 2013 and heavily-oiled and reference sites in both years (S5a Fig, Table 2). Increased Miridae abundance in the lightly-oiled areas may potentially be linked to higher amounts of nitrogen in stressed plants [57].

**S2.2 Blissidae**

The Blissid populations were similar in heavily-oiled sites and lightly-oiled sites, but significantly larger (P < 0.01) than in reference sites in 2013 (S5b Fig and Table 2). However, in 2014, Blissidae were significantly more abundant (P < 0.01) in heavily-oiled sites than in lightly-oiled and reference sites. Heavily-oiled and reference sites experienced a significant increase (P < 0.01) in Blissidae in 2014. The number of Blissidae did not significantly increase in lightly-oiled sites in 2014 (P = 0.43). Blissidae in heavily-oiled sites may have migrated from adjacent marshes because of the lowered defenses in stressed plants.

**S2.3 Coleoptera**

All sites had similar numbers of beetles (order Coleoptera), except the reference sites in 2013 (S5c Fig). After 3-4 years of oil contamination, Coleoptera may have been able to recover due to their flexible, omnivorous diets.

**S2.4 Pompiloidea**

All families of wasps in this study were included in the superfamily Pompiloidea (spider wasps). Wasps were significantly more abundant (P < 0.01) in reference sites than in heavily-oiled and lightly-oiled sites in 2013 (S5d Fig and Table 2). However, in 2014, the lightly-oiled sites had a significantly higher (P < 0.01) number of wasps than reference and heavily-oiled sites. Spider wasps predate very mobile prey (spiders). They increased along with the spiders on the studies plots.

**S2.5 Rhopalidae,**

Plant bugs from the family Rhopalidae were rare in both abundance and frequency in the samples collected. There were no significant differences among treatments (P = 0.1) and years (P = 0.2), but their interaction showed a significant difference (P = 0.03) (Table 2).

**S2.6 Lepidoptera**

Moths (order Lepidoptera) were also rare in the collected samples, with a mean value of less than one per sample (Table 2). There were no significant differences among treatments, years, and their interaction in any site (Table 2). Lepidoptera, which play an important role in plant pollination, were rare in the samples collected from all sites for both years. They were especially difficult to collect given our methods, and it was impossible to establish any effects of oil and the hurricane on such small sample sizes.
