# Supplementary figures and images for: Coupled effects of oil spill and hurricane on saltmarsh terrestrial arthropods
Source: PLoS One. 2018 Apr 11;13(4):e0194941. doi: 10.1371/journal.pone.0194941 (PMC5895010; doi:10.1371/journal.pone.0194941)

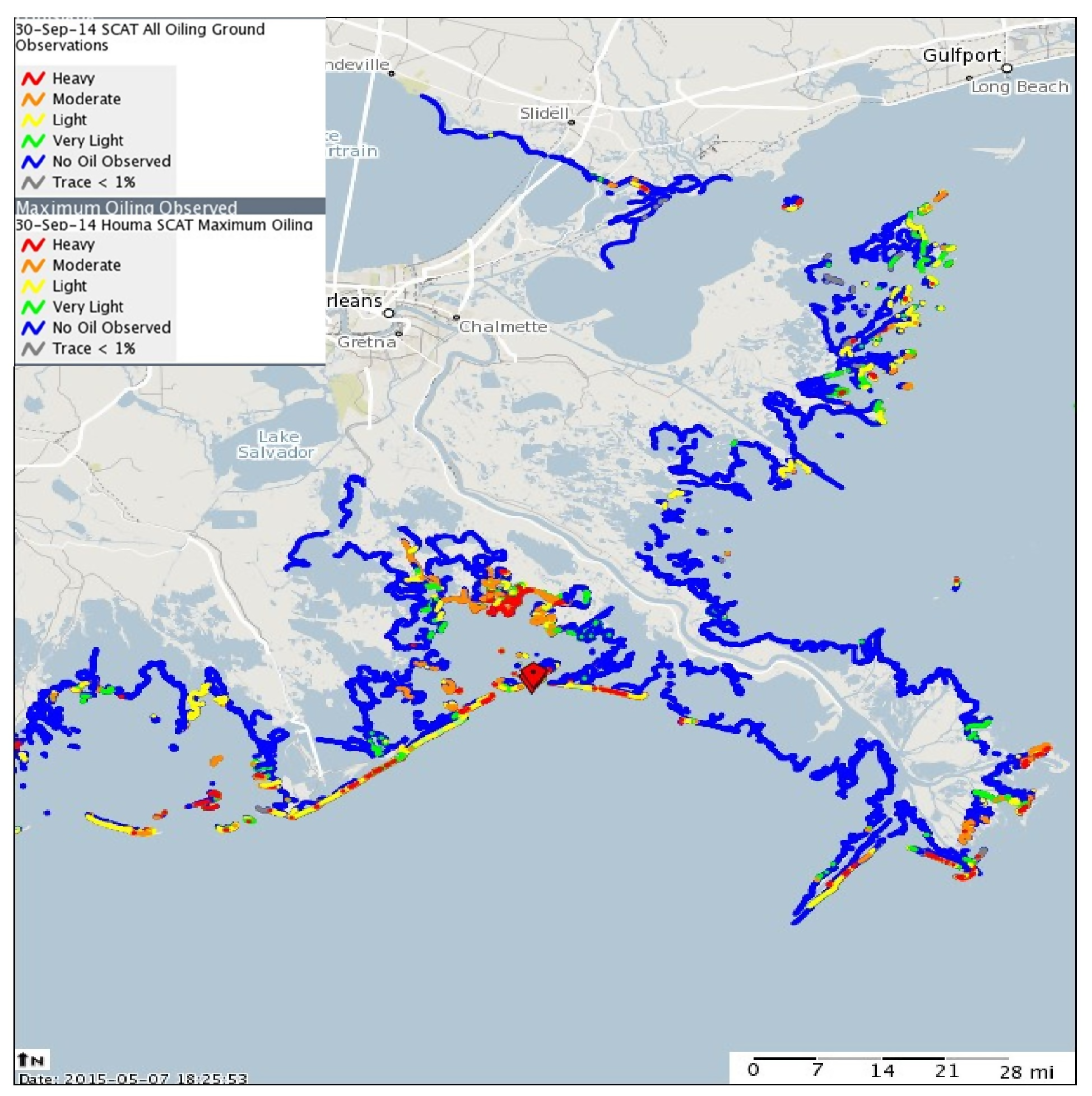

Supplement: S1 Fig — (TIFF) [file pone.0194941.s003.tiff]

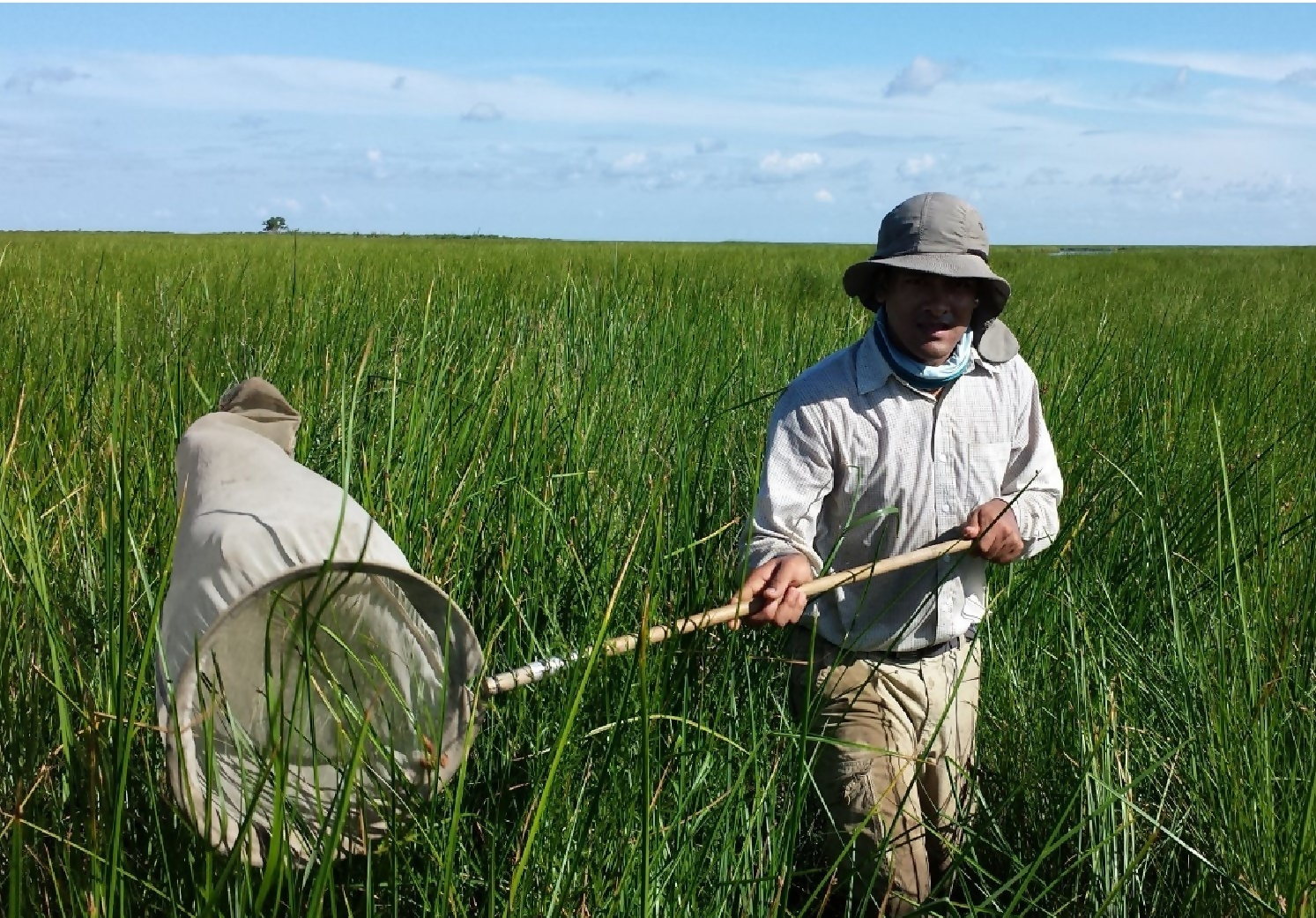

Supplement: S4 Fig — (TIFF) [file pone.0194941.s006.tiff]

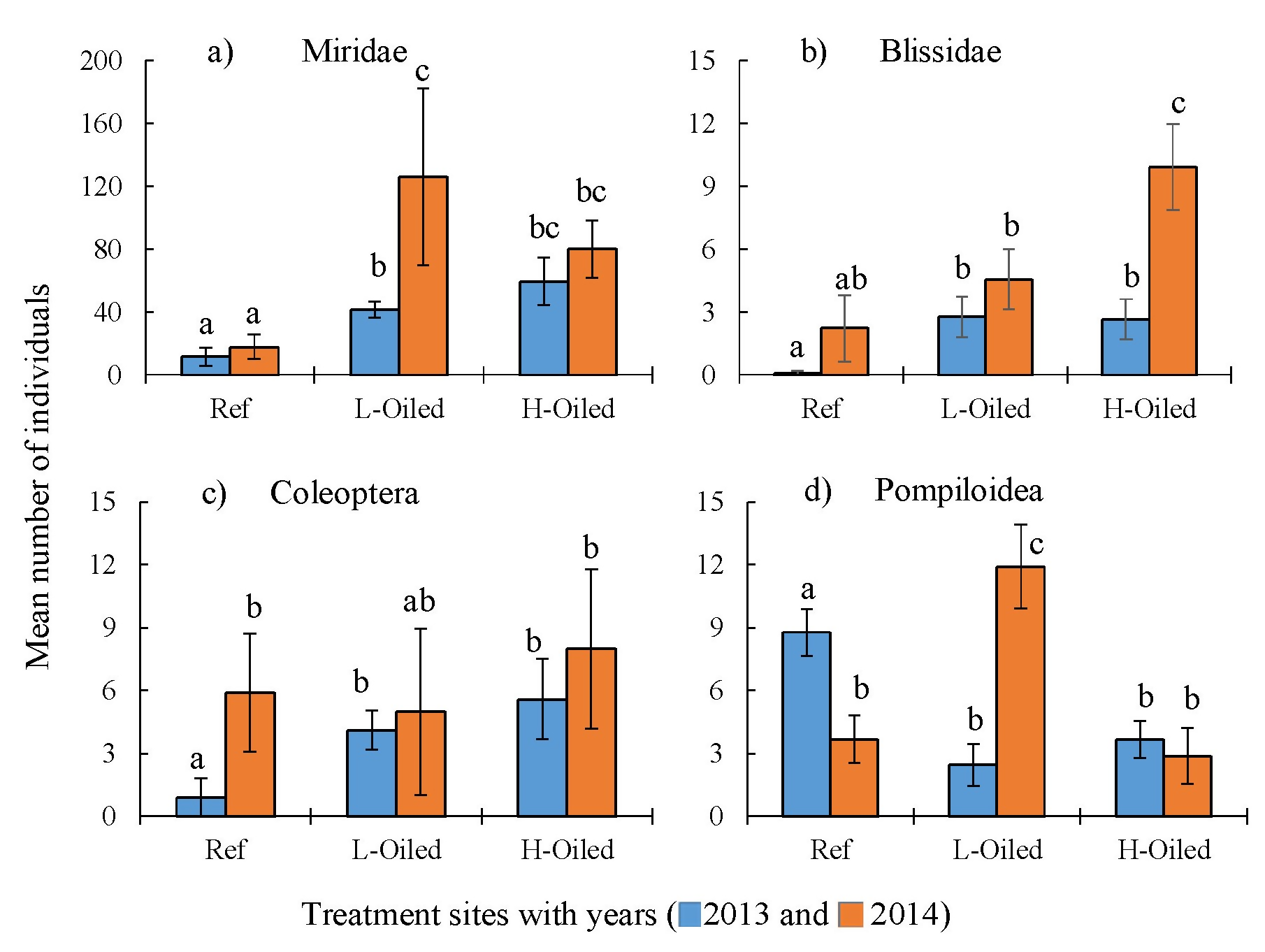

Supplement: S5 Fig — (TIFF) [file pone.0194941.s007.tiff]
